# ECG left ventricular hypertrophy in aortic stenosis: Relationship with cardiac structure, invasive hemodynamics, and long‐term mortality

**DOI:** 10.1002/clc.24155

**Published:** 2023-09-23

**Authors:** Patrizia Sager, Andreas Rusch, Lukas Weber, Alexander Breuss, Sharon Appert, Roman Brenner, Marc Buser, Peter Ammann, Hans Rickli, Micha T. Maeder

**Affiliations:** ^1^ Department of Cardiology Kantonsspital St. Gallen St. Gallen Switzerland; ^2^ University of Basel Basel Switzerland; ^3^ Department of Cardiology Kantonsspital Münsterlingen Münsterlingen Switzerland

**Keywords:** aortic stenosis, cardiac catheterization, ECG, echocardiography, left ventricular hypertrophy

## Abstract

**Background:**

In aortic stenosis (AS), left ventricular hypertrophy (LVH) is the response to pressure overload and represents the substrate for a maladaptive cascade, the so‐called AS‐related cardiac damage. We hypothesized that in AS patients electrocardiogram (ECG) LVH not only predicts echocardiography LVH but also other noninvasive and invasive markers of cardiac damage and prognosis after aortic valve replacement (AVR).

**Methods:**

In 279 patients with severe AS undergoing ECG, echocardiography, and cardiac catheterization before AVR, the Sokolow‐Lyon index, the Cornell product, the Romhilt‐Estes score, and the Peguero‐Lo Presti score were assessed.

**Results:**

The mean left ventricular mass index was 109 ± 34 g/m^2^, and 131 (47%) patients had echocardiography LVH. The areas under the receiver operator characteristics curve (AUC) for the Sokolow‐Lyon index, the Cornell product, the Romhilt‐Estes score, and the Peguero‐Lo Presti score for the prediction of echocardiography LVH were 0.59, 0.70, 0.63, and 0.65. The Peguero‐Lo Presti score had the numerically greatest AUC for the prediction of left ventricular end‐diastolic pressure >15 mmHg, mean pulmonary artery wedge pressure >15 mmHg, pulmonary vascular resistance >3 Wood units, mean right atrial pressure >14 mmHg, and stroke volume index <31 mL/m^2^. After a median follow‐up of 1365 (interquartile range: 931–1851) days after AVR only the Peguero‐Lo Presti score was significantly associated with all‐cause mortality [hazard ratio: 1.24 (95% confidence interval: 1.01–1.54); per 1 mV increase; *p* = .045].

**Conclusions:**

Among severe AS patients, the Peguero‐Lo Presti score is associated with abnormalities in cardiac structure including LVH, invasive measures of cardiac damage, and long‐term mortality after AVR.

## Graphical abstract

In 279 severe AS patients undergoing ECG, echocardiography, and cardiac catheterization, the Peguero‐Lo Presti score and three other LVH scores were assessed. The Peguero‐Lo Presti score had the greatest AUC for the prediction of key hemodynamics. After a median post‐AVR follow‐up of 1365 days only the Peguero‐Lo Presti score was significantly associated with mortality.

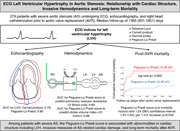

## 1 INTRODUCTION

In patients with severe aortic stenosis (AS), left ventricular hypertrophy (LVH) initially serves as a compensation for the chronic pressure overload but finally becomes maladaptive due the development of myocardial fibrosis and impairment of left ventricular diastolic function. With further disease progression left atrial dysfunction and left atrial hypertension respectively, pulmonary venous hypertension, pulmonary vascular disease, and eventually right ventricular dysfunction can occur.^1^ This detrimental cascade is referred to as AS‐related “cardiac damage,” the stage of which is prognostically important even if aortic valve replacement (AVR) is performed.^2^, ^3^

The standard 12‐lead electrocardiogram (ECG) has been used for decades to predict the presence of LVH in patients with and without AS. A large variety of ECG parameters have been proposed for this purpose.^4^ Most ECG LVH scores typically have high specificity but low sensitivity.^5^ Recently, the Peguero‐Lo Presti score as a novel ECG method to assess LVH has been described.^5^ This score has been found to have similar specificity as traditional scores but higher sensitivity in unselected patients undergoing echocardiography.^5^ In a subsequent small study, the Peguero‐Lo Presti score had a reasonable accuracy for the prediction of LVH also in patients with AS, and it also emerged as a predictor of mortality,^6^ while previous studies on the prognostic impact of ECG LVH in AS have been conflicting.^7^, ^8^

We hypothesized that LVH ECG scores are not only markers of LVH as assessed by echocardiography but also other abnormalities of cardiac structure, invasive hemodynamics reflecting AS‐related cardiac damage and mortality after AVR. In the present study, we therefore systematically assessed ECG LVH, LVH by echocardiography, other measures of cardiac structure, invasive hemodynamics, and mortality after surgical (SAVR) or transcatheter (TAVR) AVR in 279 patients with AS. Based on the above considerations we focused on the Peguero‐Lo Presti score but compared it to three other established ECG LVH scores.

## 2 METHODS

### 2.1 Study population

This is a retrospective analysis of prospectively and systematically collected data in patients with severe AS undergoing a highly standardized evaluation process before AVR in a single center between January 2011 and January 2016 (entire cohort: *n* = 503) with a post‐AVR follow‐up of several years.^9^ For this analysis, we included 279 patients undergoing left and right heart catheterization in whom a 12‐lead ECG was performed on the day before cardiac catheterization, and who had good quality transthoracic echocardiogram allowing for the assessment of left ventricular mass (LVM). Only patients with sinus rhythm were included. Patients with left bundle branch block were excluded. All patients subsequently underwent SAVR or TAVR. The study was approved by the local ethics committee. A waiver of consent was granted. We have previously reported on other hemodynamic aspects in this population.^9^, ^10^, ^11^, ^12^, ^13^, ^14^

### 2.2 ECG reading

Standard 12‐lead ECGs were acquired with 10 mm/mV calibration and a speed of 25 mm/s. All ECG were digitally stored. For the present study, all ECGs were systematically and prospectively analyzed in 2022 by a research fellow who was blinded to all clinical data including echocardiography and hemodynamics. The Peguero‐Lo Presti score was assessed as previously described. The sum of the deepest S wave plus the S wave amplitude in lead V4 was calculated, and LVH was defined according to sex‐specific cut‐offs: ≥2.3 mV for females and ≥2.8 mV for males.^5^ However, the score was also used as a continuous variable. The Sokolow‐Lyon index was calculated as S wave amplitude in V1 plus the R wave amplitude in V5 or V6 with an LVH cut‐off of ≥3.5 mV. The Cornell product was calculated as QRS duration × (R wave amplitude in aVL + S wave amplitude in V3) with a LVH cut‐off of ≥2440 mm × ms. The Romhilt‐Estes score consists of six items (voltage criteria, ST‐T abnormalities, atrial involvement, QRS axis and duration, intrinsic deflection), and LVH is considered definitive in presence of ≥5 points.

### 2.3 Echocardiography

Echocardiograms were performed by experienced cardiologists according to contemporary guidelines. Only patients with sufficient imaging quality were included. All echocardiograms were reviewed and re‐analyzed in a prospective manner in 2022 by a research fellow who was blinded to all clinical data including ECG and hemodynamics. Left ventricular dimensions were measured in the parasternal long‐axis view. LVM was calculated according to the Devereux formula: LVM = 0.8 × 1.04 × [(IVS + LVID + PWT)^3^ − LVID^3^] + 0.6, where IVS is interventricular septum thickness, LVID is left ventricular internal diameter, and PWT is posterior wall thickness.^15^ LVH was defined as left ventricular mass index (LVMI) >115 g/m^2^ in men and >95 g/m^2^ in women.^15^ Left ventricular volumes were calculated by the biplane method of disks summation technique.^15^ The basal right ventricular diameter was used as a measure of right ventricular size.^15^ Left and right atrial volumes were calculated according to the area length method.^15^

### 2.4 Cardiac catheterization

Patients underwent coronary angiography using 5 or 6 French catheters via the femoral or radial artery and right heart catheterization using 6 French Swan Ganz catheters via femoral or brachial access. The midthoracic level was used as zero reference point. Right atrial pressure, right ventricular pressure, pulmonary artery pressure, and pulmonary artery wedge pressure were measured. The wedge position was confirmed by fluoroscopy and waveform analysis. Measurements were obtained at end‐expiration, the mean pulmonary artery wedge pressure (mPAWP) was calculated over the entire cardiac cycle, and v waves were included to determine mPAWP. Cardiac output was assessed by the indirect Fick method based on blood gases, which were collected simultaneously and in duplicate from the arterial catheter and the pulmonary artery. After completion of right heart catheterization a coronary or a pigtail catheter was advanced into the ascending aorta. Systolic, diastolic, and mean aortic pressure were measured. In approximately 2/3 of the population, the aortic valve was crossed with a stiff wire, and the left ventricular end‐diastolic pressure (LVEDP) was measured using a pigtail catheter within a few minutes after the right heart catheter measurements and before coronary angiography. All pressure readings were double‐checked by the operator by manual review of the pressure tracings before they were entered into the report and used for hemodynamic calculations respectively. The transpulmonary gradient was calculated as mean pulmonary artery pressure minus mPAWP, and pulmonary vascular resistance (PVR; in Wood units, WU) was calculated as transpulmonary gradient divided by cardiac output.

#### 2.4.1 Definition of invasive hemodynamic markers of AS‐related cardiac damage

The following invasive hemodynamic markers of cardiac damage were considered: left ventricular damage (in addition to LVH): LVEDP >15 mmHg, left atrial/mitral valve damage: mPAWP >15 mmHg, pulmonary vascular damage: PVR >2 Wood units (WU) and PVR >3 WU, right ventricular/tricuspid valve damage: mean right atrial pressure (mRAP) >14 mmHg, and global cardiac damage: stroke volume index (SVI) <31 mL/m^2^. These cut‐offs are justified by the 2022 ESC/ERS guidelines on pulmonary hypertension (diagnostic or prognostic cut‐offs).^16^ We evaluated both the new (>2 WU)^16^ and the “old” (>3 WU)^17^ PVR cut‐off because the prognostic value of the 3 WU cut‐off is established in various settings.

### 2.5 Follow‐up

All patients underwent SAVR (75%) or TAVR (25%) following a median interval of 21 (12–35) days postcatheterization. Information on long‐term follow‐up was obtained by a research fellow from patients, general practitioners, and hospital or practice cardiologists. The clinical endpoint was all‐cause mortality.

### 2.6 Statistical analysis

Categorical data are presented as numbers and percentages, and continuous data are reported as mean ± standard deviation or median (interquartile range) as appropriate. Clinical characteristics and echocardiographic and hemodynamic data in patients with Peguero‐Lo Presti LVH versus those without were compared using unpaired *t*‐tests, Mann–Whitney *U* tests, or *χ* ^2^ tests as appropriate. Correlations between the Peguero‐Lo Presti score and the other ECG LVH scores with key echocardiography parameters including LVMI and invasive measures of cardiac damage were described by Pearson (all except Romhilt‐Estes score) or Spearman (Romhilt‐Estes score) correlation coefficients. Receiver operator characteristic (ROC) curves were constructed to assess the ability of the Peguero‐Lo Presti score and the other ECG LVH scores to predict echocardiography LVH, LVEDP >15 mmHg, mPAWP >15 mmHg, PVR >2 Wood WU, PVR >3 WU, mRAP >14 mmHg, and SVI <31 mL/m^2^. Cox regression was applied to describe the time‐dependent association between the Peguero‐Lo Presti score and the other ECG LVH scores and mortality. Survival rates of patients with Peguero‐Lo Presti LVH versus those without were compared using Kaplan–Meier plots and log‐rank tests. A *p* < .05 was considered statistically significant. Analyses were performed using SPSS statistical package version 25.0 (SPSS Inc.).

## 3 RESULTS

### 3.1 Study population

We studied 279 patients with a mean age of 73 ± 10 years (58% males). The mean indexed aortic valve area was 0.42 ± 0.13 cm^2^/m^2^, and the mean left ventricular ejection fraction (LVEF) was 56 ± 12%. Detailed clinical, echocardiographic, and hemodynamic characteristics of the entire study population are shown in Tables S1 and S2.

### 3.2 ECG and echocardiography LVH

**Figure 1:**
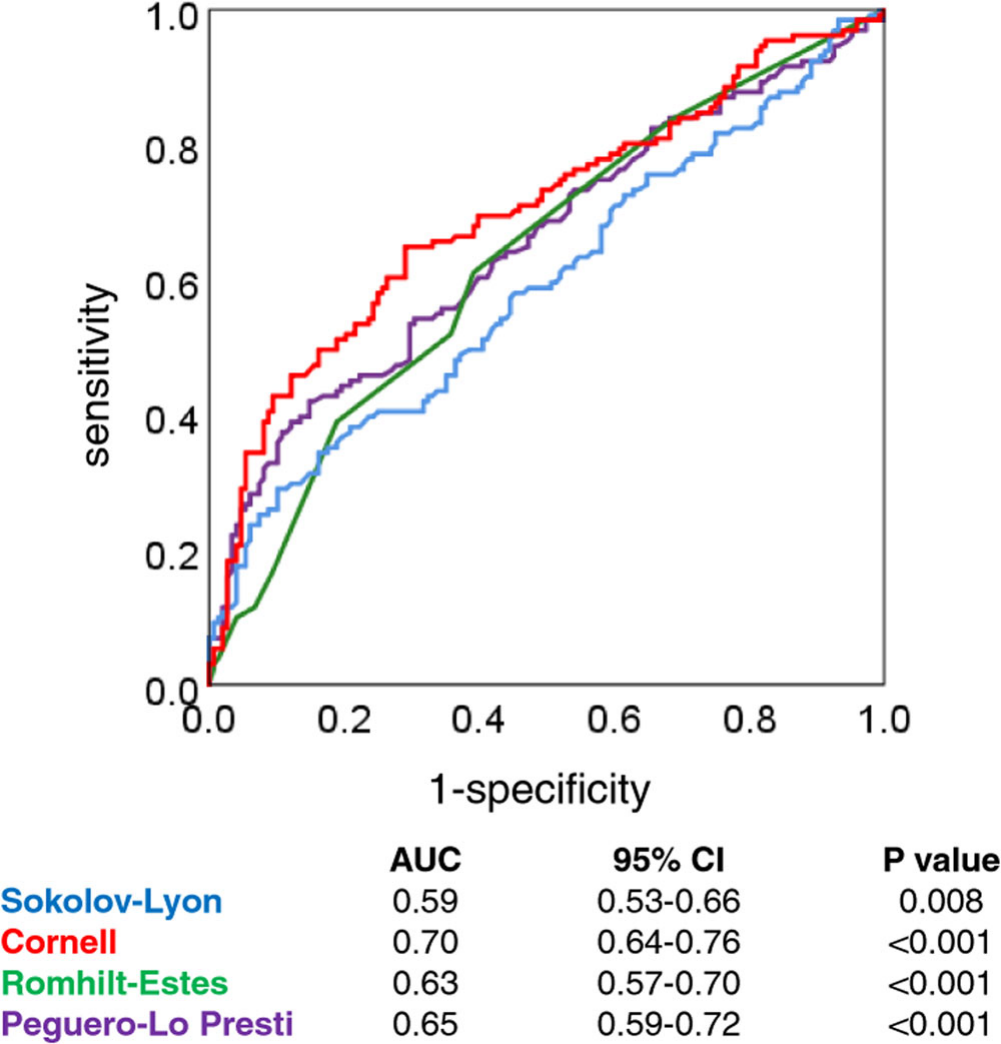
Receiver operator characteristics plots with areas under the curve (AUC) for the Sokolow‐Lyon index (blue), the Cornell product (red), the Romhilt‐Estes score (green), and the Peguero‐Lo Presti score (violet) for the prediction of left ventricular hypertrophy as determined by echocardiography. 95% CI, 95% confidence interval.

The mean LVMI was 109 ± 34 g/m^2^, and 131 (47%) patients fulfilled echocardiography LVH criteria. The correlations between all ECG scores and LVMI were statistically significant (Sokolow‐Lyon: *r* = .27; Cornell product: *r* = .42; Romhilt‐Estes: *r* = .35; Peguero Lo Presti: *r* = .42; *p* < .001 for all). The prevalence of ECG LVH according to the Sokolow‐Lyon index, the Cornell product, the Romhilt‐Estes score, and the Peguero‐Lo Presti score was 95 (34%), 83 (30%), 36 (13%), and 107 (38%). The established cut‐off for the Sokolow‐Lyon index had a sensitivity of 40% and a specificity of 72% for echocardiography LVH. The Cornell product cut‐off had a sensitivity of 47% and a specificity of 85%. The Romhilt‐Estes score had a sensitivity of 17% and a specificity of 93%. The Peguero‐Lo Presti score had a sensitivity of 50% and a specificity of 72%. The ROC curves of the four ECG LVH scores for the prediction of echocardiography LVH are shown in Figure 1. The Cornell product had nominally the highest AUC (AUC = 0.70) followed by the Peguero‐Lo Presti score (AUC = 0.65).

### 3.3 Comparison of clinical characteristics of patients with versus without Peguero‐Lo Presti ECG LVH

Patients with Peguero‐Lo Presti ECG LVH had lower body mass index and higher heart rate and B‐type natriuretic peptide, were less likely to take aspirin, and were more likely to take digoxin than patients without (Table S1). Otherwise, there were no significant differences between the groups.

### 3.4 Comparison of echocardiographic findings and invasive hemodynamics of patients with versus without Peguero‐Lo Presti ECG LVH

Patients with Peguero‐Lo Presti LVH had larger left ventricular, left atrial, right ventricular, and right atrial size but similar AS severity as expressed by the indexed aortic valve area. In addition, patients with Peguero‐Lo Presti LVH had higher mean pulmonary artery pressure, mPAWP, LVEDP, and PVR, and had lower pulmonary artery compliance, cardiac index, and SVI than those without (Table S2).

### 3.5 Relationship between ECG LVH scores and cardiac structure

Correlations between the four ECG scores and left ventricular end‐diastolic volume index, LVEF, left atrial volume index, indexed right ventricular basal diameter, and right atrial volume index are shown in Table 1. Overall, correlation were statistically significant but moderate and similar across the different ECG scores.

**Table 1:** Correlations between the different ECG LVH scores and cardiac structure.

	Sokolow‐Lyon	Cornell product	Romhilt‐Estes	Peguero‐Lo Presti
LVEDVI	*r* = .31	*r* = .28	*r* = .30	*r* = .32
	*p* < .001	*p* < .001	*p* < .001	*p* < .001
LVEF	*r* = −.16	*r* = −.27	*r* = −.29	*r* = −.25
	*p* = .01	*p* < .001	*p* < .001	*p* < .001
LAVI	*r* = .33	*r* = .34	*r* = .19	*r* = .31
	*p* < .001	*p* < .001	*p* = .007	*p* < .001
RVEDI	*r* = .27	*r* = .16	*r* = .18	*r* = .17
	*p* < .001	*p* = .01	*p* = .004	*p* = .008
RAVI	*r* = .24	*r* = .24	*r* = .20	*r* = −.29
	*p* < .001	*p* < .001	*p* = .002	*p* < .001

*Note*: Pearson correlations coefficient are shown with exception of the Romhilt‐Estes score (Spearman).

Abbreviations: LAVI, left atrial volume index; LVEDVI, left ventricular end‐diastolic volume index; LVEF, left ventricular ejection fraction; RAVI, right atrial volume index; RVEDI, indexed right ventricular diameter.

### 3.6 Relationship between ECG LVH scores and hemodynamic markers of cardiac damage

The correlations between the four ECG score and LVEDP, mPAWP, PVR, mPAP, and SVI are shown in Table 2. Correlation coefficients were numerically largest for the Peguero‐Lo Presti score except for the correlation with mRAP. In Figure S1 and Table 3, ROC curves for the prediction of LVEDP >15 mmHg (panel A), mPAWP >15 mmHg (panel B), PVR >2 WU (panel C), PVR >3 WU (panel D), mRAP >14 mmHg (panel E), and SVI <31 mL/m^2^ (panel F) by the four different ECG LVH scores are shown. For all but one parameter (PVR >2 WU), ROC curves were numerically largest for the Peguero‐Lo Presti score.

**Table 2:** Correlations between the different ECG LVH scores and key hemodynamics.

	Sokolow‐Lyon	Cornell product	Romhilt‐Estes	Peguero‐Lo Presti
LVEDP	*r* = .21	*r* = .18	*r* = .14	*r* = .27
	*p* = .004	*p* = .01	*p* = .06	*p* < .001
mPAWP	*r* = .21	*r* = .24	*r* = .20	*r* = .32
	*p* < .001	*p* < .001	*p* = .001	*p* < .001
PVR	*r* = .11	*r* = .15	*r* = .07	*r* = .23
	*p* = .07	*p* = .01	*p* = .23	*p* < .001
mRAP	*r* = .06	*r* = .06	*r* = .15	*r* = .13
	*p* = .35	*p* = .32	*p* = .01	*p* = .04
SVI	*r* = −.11	*r* = −.21	*r* = .001	*r* = −.24
	*p* = .08	*p* = .001	*p* = .91	*p* < .001

*Note*: Pearson correlations coefficient are shown with exception of the Romhilt‐Estes score (Spearman).

Abbreviations: LVEDP, left ventricular end‐diastolic pressure; mPAWP, mean pulmonary artery wedge pressure; mRAP, mean right atrial pressure; PVR, pulmonary vascular resistance; SVI, stroke volume index.

**Table 3:** Areas under the receiver operator characteristics curve (AUC) for different ECG score for the prediction of invasive measures of aortic stenosis related cardiac damage.

	AUC	95% CI	*p* value
**LVEDP** >**15** **mmHg**			
Sokolow‐Lyon	0.61	0.51–0.70	.03
Cornell	0.59	0.50–0.68	.07
Romhilt‐Estes	0.56	0.47–0.66	.21
Peguero‐Lo Presti	0.62	0.53–0.71	.01
**mPAWP** >**15** **mmHg**			
Sokolow‐Lyon	0.62	0.55–0.68	.001
Cornell	0.60	0.54–0.67	.003
Romhilt‐Estes	0.59	0.52–0.66	.01
Peguero‐Lo Presti	0.62	0.56–0.69	<.001
**PVR** >**2 WU**			
Sokolow‐Lyon	0.56	0.49–0.63	.12
Cornell	0.54	0.47–0.61	.31
Romhilt‐Estes	0.55	0.48–0.62	.14
Peguero‐Lo Presti	0.53	0.46–0.60	.45
**PVR** >**3 WU**			
Sokolow‐Lyon	0.59	0.49–0.70	.06
Cornell	0.51	0.40–0.62	.82
Romhilt‐Estes	0.59	0.49–0.69	.07
Peguero‐Lo Presti	0.60	0.50–0.70	.04
**mRAP** >**14** **mmHg**			
Sokolow‐Lyon	0.62	0.38–0.85	.30
Cornell	0.55	0.36–0.73	.68
Romhilt‐Estes	0.61	0.39–0.82	.34
Peguero‐Lo Presti	0.76	0.62–0.89	.02
**SVI** <**31** **mL/m** ^**2**^			
Sokolow‐Lyon	0.56	0.47–0.65	.15
Cornell	0.59	0.51–0.68	.03
Romhilt‐Estes	0.53	0.44–0.62	.47
Peguero‐Lo Presti	0.66	0.57–0.74	<.001

Abbreviations: CI, confidence interval; LVEDP, left ventricular end‐diastolic pressure; mPAWP, mean pulmonary artery wedge pressure; mRAP, mean right atrial pressure; PVR, pulmonary vascular resistance; SVI, stroke volume index.

### 3.7 Prognostic impact of ECG LVH

**Figure 2:**
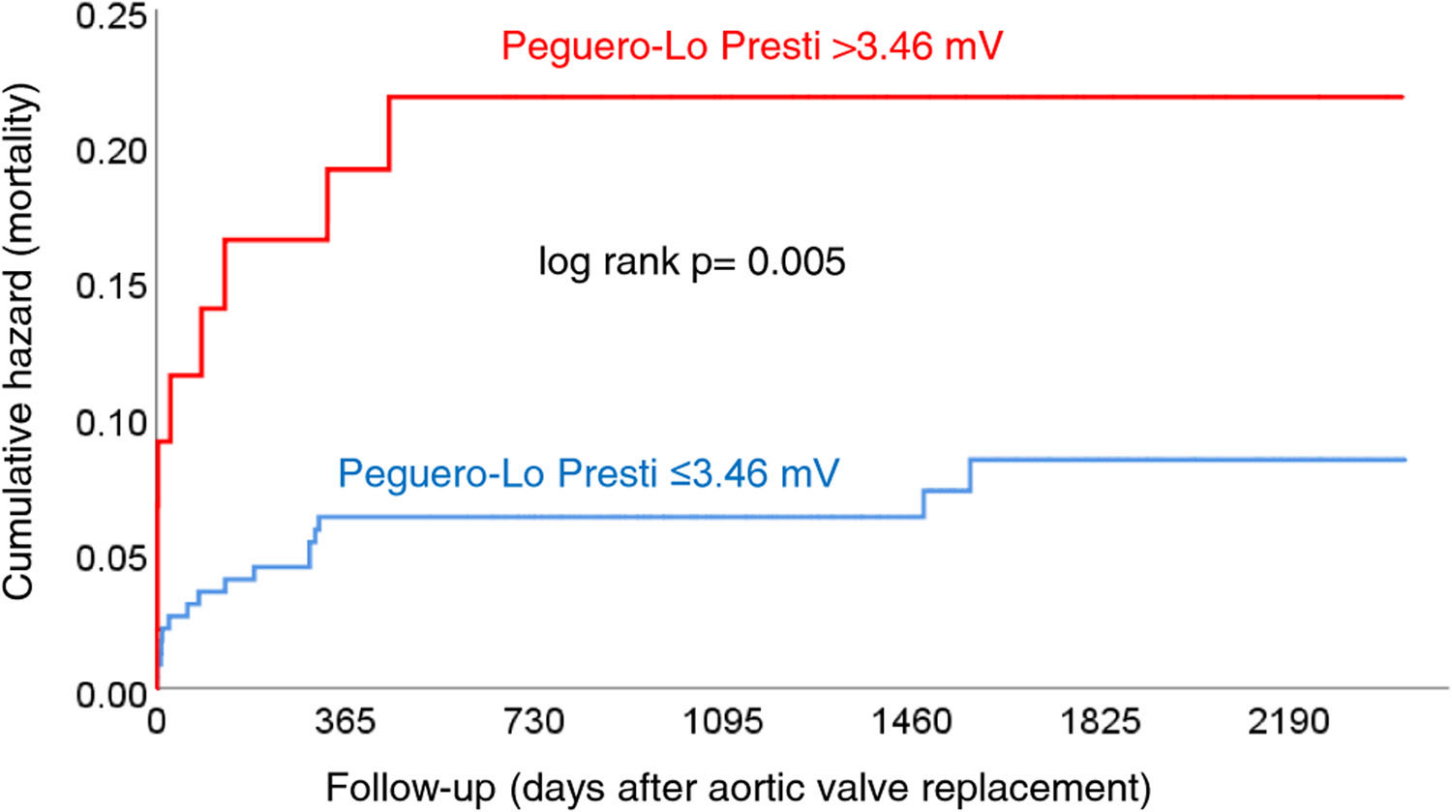
Kaplan plots showing cumulative events (mortality) for patients with Peguero‐Lo Presti score >3.46 mV versus ≤3.46 mV.

After a median follow‐up of 1365 (931–1851) days after AVR, there were 25 (9%) deaths. The Sokolow‐Lyon index [hazard ratio: 1.18 (95% confidence interval: 0.88–1.59); *p* = .01 per 1 mV increase; *p* = .28], the Cornell product [hazard ratio: 1.002 (95% confidence interval: 0.999–1.005) per 1 mm × ms increase; *p* = .23], and the Romhilt‐Estes score [hazard ratio: 1.05 (95% confidence interval: 0.89–1.24) per 1 score point increase; *p* = .55] were not associated with mortality. In contrast, the Peguero‐Lo Presti score used as a continuous variable was significantly associated with mortality [hazard ratio: 1.24 (95% confidence interval: 1.01–1.54); per 1 mV increase; *p* = .045]. Mortality between patients with and without Peguero‐Lo Presti ECG LVH according to the classical sex‐specific cut‐offs did not differ (data not shown). However, patients with Peguero‐Lo Presti score >3.47 mV (optimal cut‐off) had significantly higher mortality than those with a score ≤3.47 mV (*p* = .005; Figure 2).

Mortality in patients with echocardiography LVH was numerically higher than in those without but the difference failed to reach statistical significance (*p* = .10). Patients with PVR >2 WU versus ≤2 WU (*p* = .047), PVR >3 WU versus ≤3 WU (*p* < .001), RAP >14 mmHg versus ≤14 mmHg (*p* = .003), and SVI <31 mL/m^2^ versus ≥31 mL/m^2^ (*p* = .04) had higher post‐AVR mortality.

## 4 DISCUSSION

The present study systematically evaluating the association between four established ECG measures of LVH and cardiac structure, invasive hemodynamics, and prognosis in 279 patients with severe AS revealed several new findings: First, the accuracy of the four ECG scores including the Peguero‐Lo Presti score for the prediction of echo LVH was similar and moderate. Second, there was a statistically significant association between all ECG scores with abnormalities of cardiac structure by echocardiography and invasive measures of AS‐related cardiac damage. The AUC for most invasive cardiac damage parameters was numerically greatest for the Peguero‐Lo Presti score. Third, among the four ECG LVH scores only the Peguero‐Lo Presti score was a predictor of long‐term mortality after AVR.

The Peguero‐Lo Presti score was proposed because of higher sensitivity than the traditional scores.^5^ We could not confirm this finding. However, we studied a different population of patients with AS only. Our data are in line with study among patients with AS of variable severity (*n* = 138) revealing a sensitivity of 49% and a specificity of 84% for the Peguero‐Lo Presti score to predict LVH.^6^ In the present study, the Cornell product had the numerically highest AUC to predict LVH, i.e. a higher AUC than the Peguero‐Lo‐Presti score. In their original study, Peguero, Lo Presti and co‐workers found the opposite. However, they used the Cornell voltage criterion rather than the Cornell product.^5^

We tested the hypothesis that ECG markers of LVH not only reflect LVH per se but also AS‐related cardiac damage.^2^ We found statistically significant associations between all ECG LVH scores and cardiac structure as well as important hemodynamic parameters reflecting different aspects of cardiac damage. The Peguero‐Lo‐Presti score had the numerically highest AUC for the prediction of most pathological invasive hemodynamic parameters. In particular, the Peguero‐Lo Presti score was the best predictor of a low SVI, which formally is not a component of the cardiac damage cascade as proposed by Genereux at al.,^2^ but which can be regarded as the global extent of cardiac damage. In line with these considerations we confirmed the prognostic importance of PVR, mRAP, and SVI in this population of AS patients. We have to acknowledge that the associations between ECG LVH scores and hemodynamics were moderately strong only. Still, the observation is novel and intriguing.

Data on the prognostic value of ECG features of LVH in patients with AS have been conflicting.^7^, ^18^ A small study published in 1996 had shown that patients with severe AS on the waiting list for AVR and ECG “strain” had more events than those without.^18^ An analysis of the Simvastatin and Ezetimibe in Aortic Stenosis (SEAS) study had revealed that the Sokolow‐Lyon index and the Cornell product predicted heart failure and AVR in asymptomatic AS patients (nearly 50% with mild AS).^7^ On the other hand, in an elderly TAVR population, the absence rather than the presence of ECG LVH (by Cornell product) was found to be associated with increased mortality.^8^ In the present study, the Peguero‐Lo Presti score was linked to increased mortality after AVR while the other scores were not. In our analysis, we did not only use the sex‐specific cut‐offs for Peguero‐Lo Presti score. The ROC analysis revealed an optimal cut‐off to predict mortality that was clearly higher than the proposed cut‐offs to predict LVH with optimal sensitivity and specificity. The strength of our study is the additional availability of detailed data on cardiac structure and function and invasive hemodynamics. Overall, the Peguero‐Lo Presti was most closely related to most cardiac damage markers, and therefore the mortality data are plausible.

Currently, a number of studies using large dataset and artificial intelligence methods to evaluate the ECG for the detection of LVH or AS have been published with promising results.^19^, ^20^, ^21^ The setting of our study was different. All patients had severe AS, and we examined the value of the ECG to assess “cardiac damage” and outcome. Collectively the present data indicate that in clinical practice a simple measurement derived from the standard 12‐lead ECG is a cheap and broadly available bedside tool offering a window to the hemodynamic situation in a patient with severe AS. A high Peguero‐Lo Presti score is an important ECG finding as this is marker of an advanced stage of cardiac damage in a patient with AS.

## 5 STUDY LIMITATIONS

First, the number of patients was limited, and the data can be considered as hypothesis‐generating only. Given the availability of detailed invasive hemodynamics in combination with ECG and echocardiography data the size of the study is still attractive. Second, ECG, echocardiography, and cardiac catheterization were not performed simultaneously. However, ECG and cardiac structure by echocardiography are relatively stable, therefore, we consider this limitation not critical. Third, to assess cardiac output, we have employed the indirect Fick method, which may be subject to error, as oxygen consumption is often inaccurately estimated.^22^ This likely affects all cardiac output‐based measurements, including SVI and PVR. It must, however, be noted that this technique is routinely used in clinical practice.

## 6 CONCLUSIONS

Among patients with severe AS, the Peguero‐Lo Presti score is associated with cardiac structure including LVMI, invasive measures of AS‐related cardiac damage, and long‐term mortality after AVR.

## CONFLICT OF INTEREST STATEMENT

The authors declare no conflicts of interest.

## Supporting information

Supporting information.

Supporting information.

Supporting information.

Supporting information.

## Data Availability

The dataset of this study is not available currently because we are still working on additional analyses. Reasonable requests to obtain the data can be emailed to M.T.M. (micha.maeder@kssg.ch).
