# Supplementary figures and images for: ECG left ventricular hypertrophy in aortic stenosis: Relationship with cardiac structure, invasive hemodynamics, and long‐term mortality
Source: Clin Cardiol. 2023 Sep 23;47(1):e24155. doi: 10.1002/clc.24155 (PMC10765998; doi:10.1002/clc.24155)

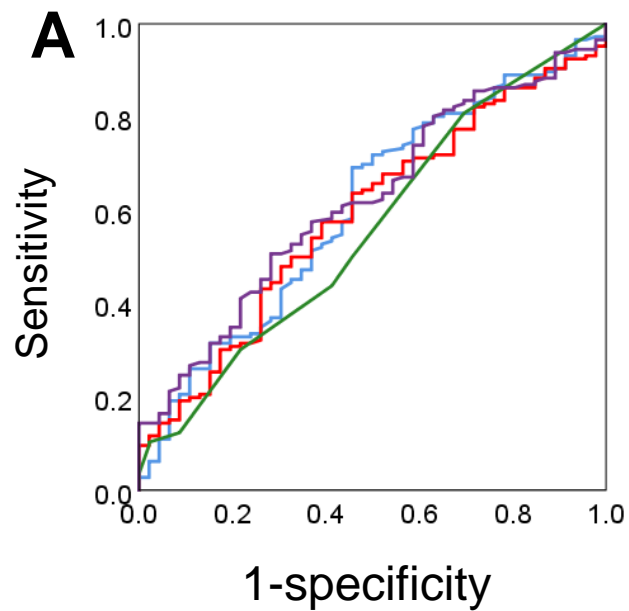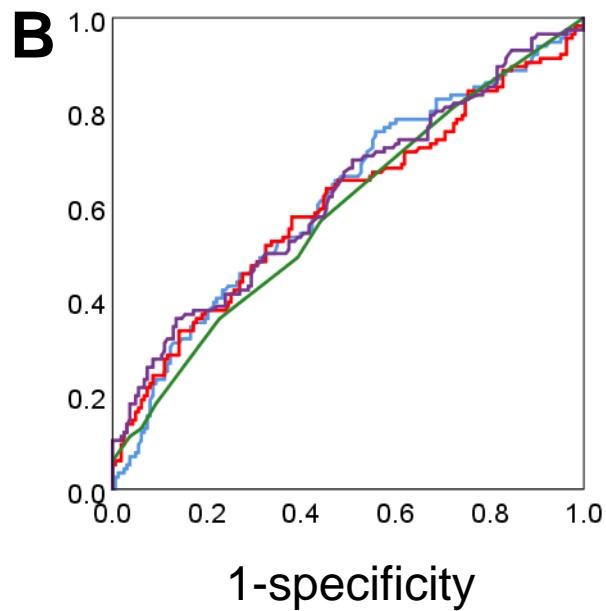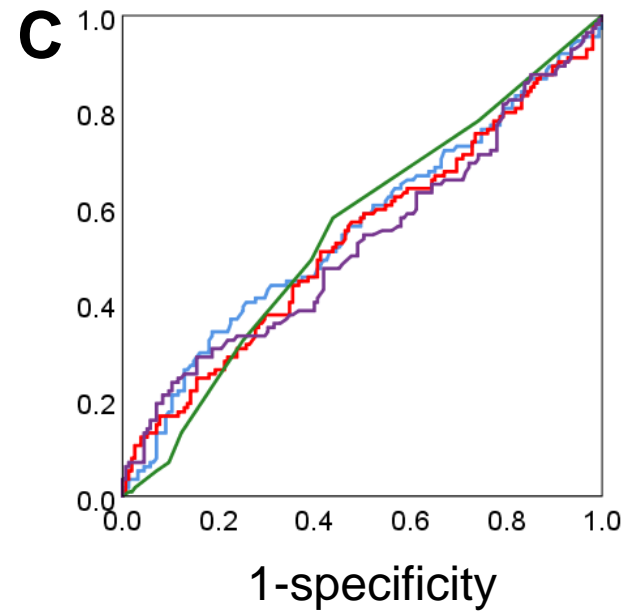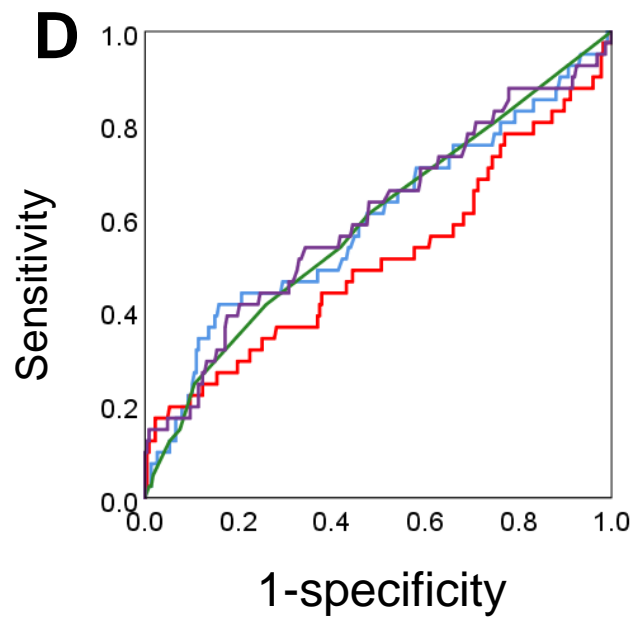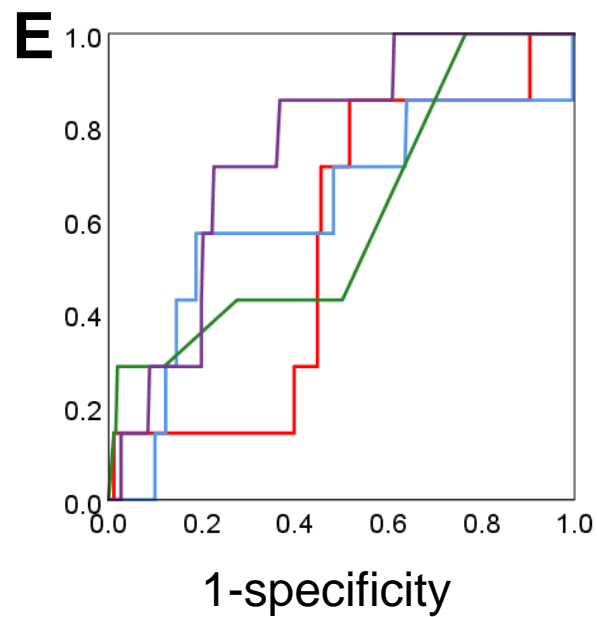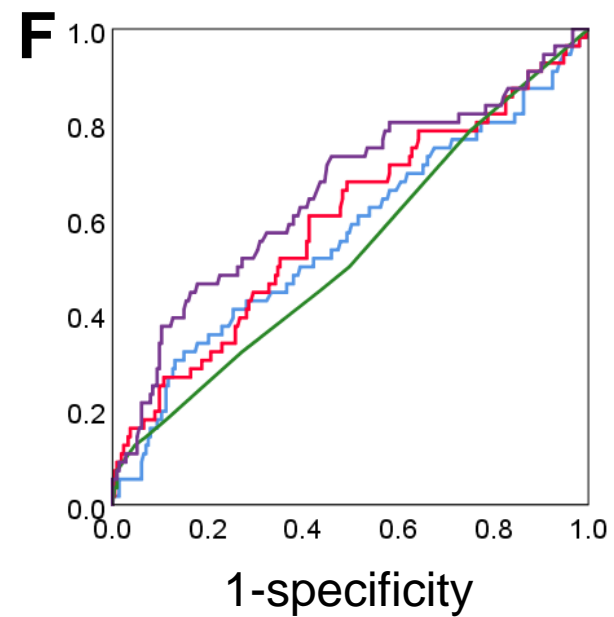

Supplement: Supplementary file 1 — Supporting information. [file CLC-47-e24155-s003.pdf]
