# Supplementary material for: ECG left ventricular hypertrophy in aortic stenosis: Relationship with cardiac structure, invasive hemodynamics, and long‐term mortality
Source: Clin Cardiol. 2023 Sep 23;47(1):e24155. doi: 10.1002/clc.24155 (PMC10765998; doi:10.1002/clc.24155)
Supplement: Supplementary file 2 — Supporting information. [file CLC-47-e24155-s001.docx]

**Supplemental** **Table S1.** Clinical characteristics of the entire study population and patients with and without Peguero-Lo Presti left ventricular hypertrophy (LVH)

|  | **All**  (n=279) | **Peguero-Lo Presti LVH**  (n=107) | **No Peguero-Lo Presti LVH**  (n=172) | **P value** |
| --- | --- | --- | --- | --- |
| Age (years) | 73±10 | 74±11 | 72±10 | 0.37 |
| Gender (male) | 167 (60%) | 60 (56%) | 107 (62%) | 0.31 |
| Body mass index (kg/m^2^) | 27.4±4.7 | 26.4±4.5 | 28.0±4.8 | 0.005 |
| eGFR (ml/min/1.73m^2^) | 74±27 | 71±26 | 76±28 | 0.32 |
| Hemoglobin (g/l) | 135±17 | 137±15 | 135±18 | 0.12 |
| Diabetes | 57 (20%) | 17 (16%) | 40 (23%) | 0.14 |
| Stroke | 13 (5%) | 5 (5%) | 8 (5%) | 0.99 |
| Chronic obstructive lung disease | 29 (10%) | 15 (14%) | 14 (8%) | 0.12 |
| FEV1 (% predicted) | 88±19 | 86±18 | 90±19 | 0.11 |
| **Heart rhythm** |  |  |  | 1.0 |
| Sinus rhythm | 279 (100%) | 107 (100%) | 172 (100%) |  |
| Atrial fibrillation | 0 | 0 | 0 |  |
| pacemaker | 0 | 0 | 0 |  |
| Heart rate (bpm) | 69±12 | 71±14 | 68±11 | 0.03 |
| **Medication** |  |  |  |  |
| Oral anticoagulation | 28 (10%) | 11 (10%) | 17 (10%) | 0.92 |
| Aspirin | 182 (65%) | 60 (56%) | 122 (70%) | 0.01 |
| Loop diuretics | 124 (44%) | 52 (49%) | 72 (41%) | 0.27 |
| Betablocker | 128 (46%) | 44 (41%) | 84 (49%) | 0.21 |
| ACEI/ARB | 140 (50%) | 53 (50%) | 87 (51%) | 0.87 |
| Digoxin | 11 (4%) | 8 (7%) | 3 (2%) | 0.02 |
| Spironolactone | 9 (3%) | 6 (6%) | 3 (2%) | 0.08 |
| B-type natriuretic peptide (ng/l) | 166 (66-408) | 359 (161-836) | 94 (51-204) | <0.001 |
| **Symptoms** |  |  |  |  |
| Dyspnea NYHA class |  |  |  | 0.15 |
| I | 61 (22%) | 21 (20%) | 40 (23%) |  |
| II | 141 (50%) | 51 (48%) | 90 (52%) |  |
| III | 67 (24%) | 28 (26%) | 39 (23%) |  |
| IV | 10 (4%) | 7 (6%) | 3 (2%) |  |
| **Mode of AVR** |  |  |  | 0.31 |
| Surgical AVR | 210 (75%) | 77 (72%) | 133 (77%) |  |
| Transcatheter AVR | 69 (25%) | 30 (28%) | 39 (23%) |  |

Data are given as numbers and percentages, mean±standard deviation, or median (interquartile range).

ACEI/ARB = angiotensin converting enzyme inhibitor/angiotensin receptor blocker; AVR = aortic valve replacement; eGFR = estimated glomerular filtration rate: FEV1 = forced expiratory volume within the first second; NYHA = New York Heart Association.
