# Supplementary material for: ECG left ventricular hypertrophy in aortic stenosis: Relationship with cardiac structure, invasive hemodynamics, and long‐term mortality
Source: Clin Cardiol. 2023 Sep 23;47(1):e24155. doi: 10.1002/clc.24155 (PMC10765998; doi:10.1002/clc.24155)
Supplement: Supplementary file 3 — Supporting information. [file CLC-47-e24155-s002.docx]

**Supplemental Table S2.** Data from echocardiography and cardiac catheterization of the entire study population and patients with and without Peguero-Lo Presti left ventricular hypertrophy (LVH)

|  | **All**  (n=279) | **Peguero-Lo Presti LVH**  (n=107) | **No Peguero-Lo Presti LVH**  (n=172) | **P value** |
| --- | --- | --- | --- | --- |
| **ECG** |  |  |  |  |
| P wave (ms) | 118±19 | 117±20 | 119±19 | 0.34 |
| QRS (ms) | 97±17 | 100±16 | 95±17 | 0.008 |
| QTc (ms) | 426±38 | 401±33 | 398±40 | 0.07 |
| Cornell product | 200 (155-260) | 273 (224-366) | 174 (131-206) | <0.001 |
| LVH criteria fulfilled | 110 (39%) | 69 (64%) | 41 (24%) | <0.001 |
| Sokolow Lyon score | 3.1±1.3 | 3.7±1.5 | 2.8±1.0 | <0.001 |
| LVH criteria fulfilled | 95 (34%) | 53 (50%) | 42 (24%) | <0.001 |
| Romhilt Estes score | 1 (1-4) | 3 (1-4) | 1 (0-3) | <0.001 |
| LVH criteria fulfilled | 36 (13%) | 20 (19%) | 16 (9%) | 0.02 |
| Peguero-Lo Presti score | 2.5±1.3 | 3.7±1.4 | 1.8±0.5 | <0.001 |
| **Echocardiography** |  |  |  |  |
| Interventricular septum (mm) | 12.8±3.6 | 13.1±3.1 | 12.6±3.8 | 0.19 |
| Posterior wall (mm) | 11.1±2.6 | 11.7±2.9 | 10.7±2.3 | 0.002 |
| Left ventricular end-diastolic diameter (mm) | 46±8 | 47±8 | 45±7 | 0.007 |
| Indexed left ventricular end-diastolic diameter | 25±4 | 26±4 | 24±4 | <0.001 |
| Left ventricular mass index (g/m^2^) | 109±34 | 124±37 | 100±30 | <0.001 |
| LVH criteria fulfilled | 131 (47%) | 66 (62%) | 65 (38%) | <0.001 |
| Left ventricular end-diastolic volume index (ml/m^2^) | 45±17 | 49±20 | 42±14 | 0.004 |
| Left ventricular end-systolic volume index (ml/m^2^) | 21±12 | 24±14 | 19±10 | 0.004 |
| Left ventricular ejection fraction (%) | 56±12 | 54±13 | 57±11 | 0.07 |
| E/e’ | 16.4±7.9 | 17.9±9.2 | 15.6±7.1 | 0.08 |
| Left atrial area (cm^2^) | 22±6 | 22±6 | 22±6 | 0.35 |
| Indexed left atrial area (cm^2^/m^2^) | 11.8±3.3 | 12.4±3.5 | 11.4±3.1 | 0.03 |
| Left atrial volume index (ml/m^2^) | 40±15 | 45±17 | 37±12 | <0.001 |
| Indexed right ventricular diameter (base; mm/m^2^) | 16±4 | 17±3 | 15±4 | 0.004 |
| Right atrial volume index (ml/m^2^) | 22±10 | 24±13 | 21±8 | 0.005 |
| Tricuspid annular plane systolic excursion (mm) | 22±5 | 21±4 | 22±5 | 0.67 |
| Estimated sPAP (mmHg) | 38±12 | 41±12 | 36±12 | 0.03 |
| Mean aortic valve gradient (mmHg) | 48±17 | 53±19 | 46±16 | 0.004 |
| Aortic valve area (cm^2^) | 0.79±0.24 | 0.75±0.26 | 0.82±0.23 | 0.03 |
| Indexed aortic valve area (cm^2^/m^2^) | 0.42±0.13 | 0.42±0.14 | 0.43±0.12 | 0.29 |
| Mitral regurgitation |  |  |  | 0.78 |
| no | 138 (49%) | 51 (48%) | 87 (50%) |  |
| mild | 113 (41%) | 43 (40%) | 70 (41%) |  |
| moderate | 23 (8%) | 11 (10%) | 12 (7%) |  |
| severe | 5 (2%) | 2 (2%) | 3 (2%) |  |
| **Coronary artery disease** |  |  |  | 0.45 |
| No coronary artery disease | 147 (53%) | 57 (53%) | 90 (53%) |  |
| 1-vessel disease | 46 (16%) | 18 (17%) | 28 (16%) |  |
| 2-vessel disease | 31 (11%) | 15 (14%) | 16 (9%) |  |
| 3-vessel disease | 55 (20%) | 17 (16%) | 38 (22%) |  |
| **Invasive hemodynamics** |  |  |  |  |
| Mean right atrial pressure (mmHg) | 6±3 | 7±4 | 6±3 | 0.50 |
| Right ventricular end-diastolic pressure (mmHg) | 8±4 | 9±4 | 8±3 | 0.63 |
| sPAP (mmHg) | 38±14 | 41±16 | 37±12 | 0.005 |
| dPAP (mmHg) | 15±7 | 17±9 | 14±6 | <0.001 |
| mPAP (mmHg) | 25±10 | 27±12 | 23±9 | 0.002 |
| mPAWP (mmHg) | 15±8 | 18±9 | 14±7 | <0.001 |
| Transpulmonary gradient (mmHg) | 9±5 | 9±5 | 9±4 | 0.52 |
| Pulmonary vascular resistance (Wood units) | 2.1±1.3 | 2.3±1.6 | 1.9±1.1 | 0.02 |
| Pulmonary artery compliance (ml/mmHg) | 3.5±1.7 | 3.0±1.5 | 3.8±1.8 | 0.001 |
| Left ventricular end-diastolic pressure (mmHg) n=192 | 21±8 | 23±8 | 20±8 | 0.01 |
| Systolic aortic pressure (mmHg) | 147±26 | 147±28 | 147±25 | 0.83 |
| Diastolic aortic pressure (mmHg) | 68±12 | 68±12 | 68±11 | 0.71 |
| Mean aortic pressure (mmHg) | 99±14 | 97±15 | 99±14 | 0.31 |
| Arterial oxygen saturation (%) | 95 (94-97) | 95 (94-97) | 95 (94-97) | 0.78 |
| Mixed venous oxygen saturation (%) | 69 (65-72) | 68 (64-71) | 70 (67-73) | 0.001 |
| Cardiac output (l/min) | 4.8±1.0 | 4.5±0.9 | 5.0±1.1 | <0.001 |
| Cardiac index (l/min/m^2^) | 2.6±0.5 | 2.5±0.5 | 2.6±0.5 | 0.021 |
| Stroke volume (ml) | 70±20 | 66±19 | 75±19 | <0.001 |
| Stroke volume index (ml/m^2^) | 39±10 | 36±10 | 40±10 | 0.004 |

Data are given as numbers and percentages, mean±standard deviation, and/or median (interquartile range).

dPAP = diastolic pulmonary artery pressure, E/e’ = ratio of peak early mitral inflow velocity to peak early mitral annular velocity, mPAP = mean pulmonary artery pressure; mPAWP = mean pulmonary artery wedge pressure, sPAP = systolic pulmonary artery pressure.
