# Supplementary material for: ECG left ventricular hypertrophy in aortic stenosis: Relationship with cardiac structure, invasive hemodynamics, and long‐term mortality
Source: Clin Cardiol. 2023 Sep 23;47(1):e24155. doi: 10.1002/clc.24155 (PMC10765998; doi:10.1002/clc.24155)
Supplement: Supplementary file 4 — Supporting information. [file CLC-47-e24155-s004.docx]

**Legend Supplemental Figure**

**Supplemental Figures S1.** Receiver operator characteristics plots with areas under the curve (AUC) for the Sokolow-Lyon index (blue), the Cornell product (red), the Romhilt-Estes score (green), and the Peguero-Lo Presti score (violet) for the prediction of left ventricular end-diastolic pressure >15 mmHg (panel A), mean pulmonary artery wedge pressure >15 mmHg (panel B), pulmonary vascular resistance (PVR) >2 WU (panel C), PVR >3 WU (panel D), mean right atrial pressure >14 mmHg (panel E), and stroke volume index <31 ml/m^2^ (panel F). The exact AUC numbers are shown in Table 3.
